# Trust, Trustworthiness, and the Future of Medical AI: Outcomes of an Interdisciplinary Expert Workshop

**DOI:** 10.2196/71236

**Published:** 2025-06-02

**Authors:** Melanie Goisauf, Mónica Cano Abadía, Kaya Akyüz, Maciej Bobowicz, Alena Buyx, Ilaria Colussi, Marie-Christine Fritzsche, Karim Lekadir, Pekka Marttinen, Michaela Th Mayrhofer, Janos Meszaros

**Affiliations:** 1 Department of ELSI Services and Research Biobanking and Biomolecular Resources Research Infrastructure Consortium Graz Austria; 2 2nd Department of Radiology Gdańsk Medical University Gdansk Poland; 3 Institute of History and Ethics in Medicine TUM School of Medicine and Health Technical University of Munich Munich Germany; 4 Department of Science, Technology and Society (STS) School of Social Sciences and Technology Technical University of Munich Munich Germany; 5 Institució Catalana de Recerca i Estudis Avançats (ICREA) Department of Mathematics and Computer Science Universitat de Barcelona Barcelona Spain; 6 Department of Computer Science Aalto University Espoo Finland; 7 Division of Clinical Pharmacology and Pharmacotherapy Department of Pharmaceutical and Pharmacological Sciences KU Leuven Leuven Belgium

**Keywords:** artificial intelligence, trust, trustworthy AI, medicine, ethics of AI, interdisciplinarity, stakeholder engagement, human-centered AI

## Abstract

Trustworthiness has become a key concept for the ethical development and application of artificial intelligence (AI) in medicine. Various guidelines have formulated key principles, such as fairness, robustness, and explainability, as essential components to achieve trustworthy AI. However, conceptualizations of trustworthy AI often emphasize technical requirements and computational solutions, frequently overlooking broader aspects of fairness and potential biases. These include not only algorithmic bias but also human, institutional, social, and societal factors, which are critical to foster AI systems that are both ethically sound and socially responsible. This viewpoint article presents an interdisciplinary approach to analyzing trust in AI and trustworthy AI within the medical context, focusing on (1) social sciences and humanities conceptualizations and legal perspectives on trust and (2) their implications for trustworthy AI in health care. It focuses on real-world challenges in medicine that are often underrepresented in theoretical discussions to propose a more practice-oriented understanding. Insights were gathered from an interdisciplinary workshop with experts from various disciplines involved in the development and application of medical AI, particularly in oncological imaging and genomics, complemented by theoretical approaches related to trust in AI. Results emphasize that, beyond common issues of bias and fairness, knowledge and human involvement are essential for trustworthy AI. Stakeholder engagement throughout the AI life cycle emerged as crucial, supporting a human- and multicentered framework for trustworthy AI implementation. Findings emphasize that trust in medical AI depends on providing meaningful, user-oriented information and balancing knowledge with acceptable uncertainty. Experts highlighted the importance of confidence in the tool's functionality, specifically that it performs as expected. Trustworthiness was shown to be not a feature but rather a relational process, involving humans, their expertise, and the broader social or institutional contexts in which AI tools operate. Trust is dynamic, shaped by interactions among individuals, technologies, and institutions, and ultimately centers on people rather than tools alone. Tools are evaluated based on reliability and credibility, yet trust fundamentally relies on human connections. The article underscores the development of AI tools that are not only technically sound but also ethically robust and broadly accepted by end users, contributing to more effective and equitable AI-mediated health care. Findings highlight that building AI trustworthiness in health care requires a human-centered, multistakeholder approach with diverse and inclusive engagement. To promote equity, we recommend that AI development teams involve all relevant stakeholders at every stage of the AI lifecycle—from conception, technical development, clinical validation, and real-world deployment.

## Introduction

Trustworthiness has become a key requirement in the development and application of ethical artificial intelligence (AI). This is highlighted by the European Commission High-Level Expert Group on AI (AI HLEG) Guidelines for Trustworthy AI [1] and emphasized by the World Health Organization (WHO) [2] for effective AI integration in health care. In addition, cross-cutting initiatives such as FUTURE-AI (Fairness, Universality, Traceability, Usability, Robustness, and Explainability–artificial intelligence) aim to guide AI developments toward trustworthiness, focusing on principles of fairness, universality, traceability, usability, robustness, and explainability [3]. The discourse on trustworthy AI often focuses on defining the conditions under which it can be achieved. Scholars are developing practical approaches for realizing trustworthy AI through guidelines or regulations [4], and some institutions even offer certification possibilities (eg, IEEE CertifAIEd). Such efforts neglect that ethical AI cannot be guaranteed solely through following principles [5,6], or that trustworthy AI cannot only be defined in terms of technical conditions and solutions [7], such as fairness objectives [8]. Indeed, some authors have challenged the idea of achieving trustworthy AI by merely meeting technical criteria and have argued that trusting in AI is based on more human than technical aspects [9,10]. Besides, guidelines on the implementation of “trustworthy AI” in medicine lack consensus on what defines a user’s trust in AI [11].

The significance of the discourses around trust in AI and the trustworthiness of AI within the scientific discussion on ethical AI is rarely reflected in its conceptualization [12,13], which is crucial as AI “can increase systemic risks of harm, raise the possibility of errors with severe consequences, and amplify complex ethical and societal issues” [14]. In scientific literature, the concepts of “trust” and “trustworthiness” are often used interchangeably, despite repeated efforts to define them [15,16]. A more thorough analysis is needed on the situatedness of trust in medical AI and the trustworthiness of medical AI, given that trust plays a critical role in situations of risk, vulnerability, and uncertainty, circumstances frequently encountered in the medical context. In this context, trust needs to be understood as a complex relational concept that involves several trustor-trustee relationships, such as trust in technology, institutions, and persons, for example, scientists who trust each other, patients who trust scientists, and health care professionals [17,18].

Trustworthiness of medical AI also depends on how certain challenges are being managed: for instance, biases in the training data that lead to biases in algorithms, lack of transparency or explainability in how an AI system decides, securing sensitive data such as medical data, as well as ensuring human oversight and continuous monitoring [19]. Against the backdrop of evidence showing that algorithms employed in health care can encode, reinforce, and exacerbate existing inequalities within the health care system [20], which poses a particular risk to vulnerable patients [21], identifying and mitigating biases, especially racial and gender biases [22], is key for trust [23,24]. Failure to do so could erode public trust in health systems and hinder the adoption of AI systems in health care [25]. Consequently, addressing bias must go beyond the prevailing focus on computational factors and fairness of machine learning algorithms and must take all forms of potential bias into account, including human, institutional, and societal factors [26].

This viewpoint article presents an interdisciplinary approach to analyzing trust in AI and trustworthy AI within the medical context and proposes a practice-oriented conceptualization. Adopting a multidisciplinary understanding of trust and trustworthiness, it builds on the outcomes of a workshop that brought together experts from various disciplines involved in the development and application of medical AI, particularly in oncological imaging and genomics. The aim was to address analytical gaps and explore the concepts of trust and trustworthiness. This interdisciplinary and contextualized standpoint informs the analysis of trust and trustworthiness in medical AI from knowledge production to technology development and use. In doing so, the article puts an emphasis on the social and legal conceptualizations of trust and their implications for trustworthy AI in medicine.

## Trust in Humanities and Social Sciences

While there is a plethora of literature on trust and trustworthiness of AI [15,16,27] and in medicine in particular [11,28-30], a deeper conceptualization of the terms used is needed for specific practices in medicine and health care.

Across different philosophical approaches (eg, virtue ethics or deontological ethics), trust is considered interpersonal and relational, involving risk and moral responsiveness to patients’ vulnerability. Carter [31] notes that illness induces feelings of vulnerability in patients and caregivers, highlighting the need for a mature exploration of trust, distinct from related concepts like reliance, confidence, or faith. Baier [32] defines trust as a mix of reliance, confidence, and dependence. Reliance involves trusting another’s competence, while trust involves depending on their goodwill. Baier asserts that everyone is interdependent, relying on others for care. This aligns with more recent literature, which emphasizes our constitutive vulnerability and interdependence [33-36]. Baier [32] also highlights the role of power relations, stressing that morality requires trust and consideration of potential exploitation in unequal power dynamics.

Sociological perspectives on trust, particularly those of Giddens and Luhmann [37], explore the dynamics of institutional and interpersonal trust. Institutional trust, such as in the health care system, is influenced by trust in its representatives, like doctors. Giddens emphasizes the role of the health care professional’s appearance and professionalism in shaping patient expectations and legitimizing the medical system. He asserts that trust in “flesh-and-blood” representatives informs trust in the system and is necessary to manage partial understanding due to uncertainty. Trust is not needed in situations with complete knowledge. Luhmann, however, views trust as a medium that reduces social complexity, enabling interactions within and by the system. He distinguishes between trust and confidence: trust involves past experiences and perceived risks, whereas confidence relies solely on expectation without considering alternatives [37].

Trust has been a central topic in the social sciences’ examination of science and knowledge production, emphasizing the interplay between individuals and social institutions. Understanding trust requires recognizing its foundation in social relations and exploring its interactive nature, including the role of technology and the importance of tacit, embodied, and situated knowledge [38,39]. These elements incorporate social, practical, human, and bodily factors into the comprehension of knowledge production and mutual trust.

Science and technology studies (STS) conceptualize trust in relational terms, particularly focusing on the publics of science and technology. A significant shift has been the critique of the “deficit model,” which assumes public mistrust stems from a lack of understanding [40,41]. This critique has led to a transformation in building trust through communicative practices, moving from one-directional communication to models of engagement that recognize the diversity of publics. Despite this, critics note the persistent reemergence of the deficit model even in newer efforts [42]. Concerns about emerging technologies’ risks and uncertainties highlight the need to reflect on modernity’s inherent crises and their relation to policymaking, accountability, transparency, and expertise [43]. STS scholars emphasize the importance of considering various forms of expertise, including lay expertise [41,44,45]. Cases of public acceptance or rejection of technologies like nuclear energy, nanotechnology, and genetically modified organisms provide insights into contemporary AI developments [46,47]. One-way communication does not automatically increase public trust; technologies often carry risks not immediately apparent to technologists, necessitating reflexivity [48] and a global perspective [49]. Hence, building trust would require transparent, adaptable governance systems and strong trust relations between individuals and institutions, respecting the variety of expertise.

In examining the intersection of trust and AI within medicine, it may be relevant to translate insights into trust offered by the humanities and social sciences into this emerging field. Table 1 presents a synthesis of classical approaches to trust outlined above, highlighting their relevance and application of AI in health care.

**Table 1:** Translation of selected classical approaches to trust in the humanities and social sciences into the field of AI^a^ and medicine.

Approach	Translation
Trust as a relational and moral concept (Carter [31], Baier [32])	Trust in AI is viewed as a relationship in which ethical behavior, transparency, and accountability are essential. Trust is not only about the system’s performance but also about the intentions and actions of those who design and manage AI systems.
Trust involving risk and moral responsiveness (Pellegrino & Thomasma [50], Emanuel & Emanuel [51])	Trust in AI involves stakeholders assuming risks, such as data privacy risks or misdiagnoses, with the expectation that AI systems will function ethically, respecting user autonomy and promoting well-being.
Foundational trust in doctor-patient relationships (Kittay & Meyers [52])	In AI-enhanced health care, trust is crucial for integrating AI tools into patient care, affecting how patients perceive and cooperate with AI-driven diagnostics and treatment plans.
Institutional trust (Giddens, Luhmann in Meyer et al [37])	Trust in AI within institutions depends on the trustworthiness of those who deploy and manage AI systems, influencing public and professional trust in the technology’s utility and safety.
Dynamic trust shaped by social relations, technology, and experience (Collins [39], Haraway [38], Meyer et al [37])	Trust in AI is dynamic, shaped by ongoing interactions between users, developers, and AI systems. This includes how technology adapts to social expectations and how it is embedded in social practices.

^a^AI: artificial intelligence.

## Legal Perspectives on Trust and Trustworthiness

In legal studies, the concept of trust is often overlooked [53,54] except for “trust law” in common law systems, which involves a fiduciary relationship where a “trustor” transfers property or rights to a trustee [55]. “Good faith” and “due diligence” embody elements of trust, implying confidence in a person or entity. For example, in contract law, good faith is essential, presuming honest and fair dealings between parties. Thus, trust is implicitly part of good faith. When trust is violated, liability rules restore balance [56,57].

Some authors argue that the law substitutes for trust [58] or emerges where trust is lacking [59]. Law’s coercive and controlling nature can crowd out trust, especially “personal trust” in individuals or firms, which could mean that legislation cannot effectively promote trust. Instead, the law can define and establish rules for trustworthy behaviors. Greco [60] challenges the idea that law and trust are incompatible, arguing that law can play a constructive role in fostering trust and serving as a bridge between citizens and institutions, thereby generating and sustaining social trust. In other words, the law not only imposes rules but also helps people feel safe, respected, and thus more inclined to trust the system in which they live. From a legal philosophy perspective, trust underpins the law’s existence: human relationships, which the law regulates, rely on trust. Sanctions rebuild violated trust, and rules emerge from trust, with the expectation of compliance. Thus, trust is foundational to the law.

Like trust, trustworthiness is not inherently a legal concept. In the context of AI, the European Union (EU) has emphasized trustworthiness, specifically through the concept of “trustworthy AI” rather than focusing on “trust” itself. The Ethics Guidelines for Trustworthy AI by the AI HLEG [1] define AI as being “lawful, ethical, and robust.” Smuha et al [61] indicate three pillars of “Legally Trustworthy AI”: (1) responsibility allocation, where the regulation appropriately assigns accountability for the harms and wrongs resulting from AI systems; (2) a consistent legal framework, where the regulation establishes and maintains a unified legal structure accompanied by effective and legitimate enforcement mechanisms to secure and uphold the rule of law; and (3) democratic deliberation, where the regulation places democratic discussion at its center, ensuring public participation and other information rights. These pillars were crucial when the EU AI Act was drafted, aiming to establish a robust legal framework to foster the development of secure, trustworthy, and ethical AI. The EU AI Act, which entered into force on August 1, 2024, establishes a comprehensive regulatory framework for AI within the EU [62]. The Act highlights that “in the health sector where the stakes for life and health are particularly high, increasingly sophisticated diagnostics systems and systems supporting human decisions should be reliable and accurate” (Recital 47). The AI Act takes a human-centric approach and recalls the guidelines for trustworthy AI by the AI HLEG (see Recital 27), namely: human agency and oversight, technical robustness and safety, privacy and data governance, transparency, diversity, nondiscrimination and fairness, societal and environmental well-being, and accountability. It states that these principles should be applied in the design and use of AI models and should serve as a basis for the drafting of codes of conduct under the Regulation.

The AI Act mentions “trust” or “trustworthiness” 29 times, underscoring the significance of building trust in AI technologies. The health sector is significantly impacted by the EU AI Act, particularly due to the inclusion of AI systems. Moreover, the AI Act states that AI systems classified as medical devices under Medical Device Regulation (MDR) [63] or In Vitro Device Regulation [64]—and thus those AI systems used as medical devices—must comply with both the AI Act and existing medical device regulations. There are exemptions only for AI systems used exclusively for scientific research and premarket product development. In light of the AI Act, therefore, it seems that most commercial AI-enabled medical devices on the market used in radiology, being classified as Class IIa and up, under the MDR [65], are classified as high risk under the AI Act [66].

These developments and the prominence that “trust” and “trustworthiness” have achieved in law and policy, while being scarcely defined, highlight the need for a comprehensive analysis, particularly from an interdisciplinary and practical perspective. Toward this end, this paper advances the discussion by examining how the EU AI Act gives legal meaning to the otherwise vague concept of “trustworthy AI.” It highlights how trust is not only a technical or ethical aspiration, but a legal objective shaped by rules on accountability, transparency, and human oversight, especially in health care settings.

## Synthesizing Interdisciplinary Knowledge

To make better sense of issues around ethical AI as well as understandings and conditions of trust and trustworthy AI in medicine, we organized a 2-day workshop with 14 academic experts in data science, law, medicine, ethics, social science, and philosophy, which took place on May 5-6, 2022, in Berlin, Germany. The workshop participants were researchers representing four large European projects (EuCanImage, INTERVENE, Bigpicture, and BIOMAP) and other experts who contributed perspectives from philosophical and policy research. The focus was on how to implement the ethics of AI, drawing from experiences in oncological imaging and genomics. The workshop combined presentations and discussions on ethical, legal, societal, technical, and clinical aspects of medical AI.

Inspired by focus group and stakeholder engagement methods [67-69], the workshop synthesized interdisciplinary inputs on explainability and trustworthiness of AI solutions, clinical applications in cancer imaging, polygenic risk score generation and use, biases in training and calibration of AI models, and algorithmic impact assessment, as well as moderated discussions between the experts. The workshop discussions were recorded based on the informed consent of all participants, and key discussion points were put on digital sticky notes and related to each other on a digital mural during the workshop.

To deepen these discussions, the workshop participants were invited to partake in an online questionnaire consisting of five open-ended questions to share further thoughts and more specific insights into their field of expertise. Since trust and trustworthiness emerged as a crucial topic in the workshop discussions, the questions were on field-specific definitions of trust in the context of AI, criteria of trustworthy AI applications, for trusting relationships involving AI, and for trustworthiness as a valuable principle.

Aspects of trustworthy AI in medicine were also guiding the analysis of the workshop discussions and questionnaire responses. The analysis team (MG, KA, MCA) interpreted the workshop discussions and questionnaire answers in several meetings and conducted thematic analysis [70,71] to unpack the requirements, conditions, and challenges around trustworthy AI that are presented in the following sections of this article.

While topics like bias and fairness—often central and technically-focused within AI ethics discussions—have received considerable attention, we aim here to highlight equally critical yet more socially and practically oriented themes, specifically the importance of knowledge and human involvement. These dimensions, as detailed by practitioners, offer deeper insights into real-world challenges within the medical field.

## The More We Know, the More We Trust?

In the scientific discourse on AI, the trustworthiness of an AI system is closely tied to the requirement that the decision-making process must be transparent, explainable, and understandable, to allow medical professionals to make informed decisions [72]. This should include that AI systems communicate their limitations, for example, their inability to provide an accurate answer for a given patient or, in general, having a greater accuracy for certain patient groups, as well as include proper uncertainty quantification for their results [73]. This allows doctors to assess the appropriateness of using the AI tool for specific patients.

The need for transparency resonates with the fact that medical knowledge production inherently involves a degree of uncertainty. In scientific knowledge production, organized skepticism is part of the ethos of science [74]; however, the temporalities of clinical practice and scientific knowledge production differ. For AI, providing comprehensive explanations of internal processes may often be unrealistic [75]. Besides, striving for more explainable AI systems could reduce their efficacy, depending on the explainability techniques applied. For example, if a model is designed to be explainable from the outset using a simple model structure, like a linear additive model, its predictive performance could be compromised. On the other hand, even a simple model based on causal relationships verified through careful interpretation and domain knowledge may be robust and generalizable [76], but could be difficult to achieve for complex data types such as images or text. Hence, the pursuit of trustworthy AI raises questions about the balance between transparency and complexity.

Workshop outcomes indicate that what makes a difference to the trustworthiness of medical AI systems is not necessarily more, but more meaningful, situation- and user-oriented information as well as a good balance between knowledge and acceptable uncertainty. Experts highlighted that it is essential to ensure that the AI tool functions exactly as it is expected. Rather than detailed insights into the inner workings of an AI system, clarifying what knowledge is needed by which actors and areas of application has been emphasized. For instance, in the clinical context, it was deemed essential to know how the AI tool was built, eg, the type of dataset that was used to train the algorithm, its limitations and biases, the accuracy of the performance, but also the uncertainty of the outcome.

User-oriented knowledge takes several information needs of different stakeholders into account, such as developers, users, and patients, and these vary. For instance, developers benefit from multiple explainability techniques to identify biases to make sure that the predictions are not based on spurious correlations in the training data. In this respect, medical doctors might benefit from highlighting the part of the x-ray image that indicates the presence of cancer. From the patient’s perspective, explainability is of interest primarily through the attending physician’s judgment and experience. Patients may trust their physician more than they rely on the explainability of the AI system.

The requirement for specific types of knowledge reflects the relational aspects associated with explainability and AI trustworthiness. Several quantitative and qualitative explainability methods are used in AI development to understand why and how AI systems make specific predictions [77]. However, it is crucial to involve physicians during the tool development and testing stages in a human-in-the-loop manner. As domain experts, physicians use explainability tools to verify the accuracy of system predictions based on correct premises.

Explainability methods are essential for AI-based software as a medical device. Daily operation of the tool does not necessarily require explainability, though it becomes useful if medical professionals disagree with its predictions. In such cases, explainability algorithms help verify the prediction premises. Local visualization methods like heat maps or attention maps, supported by uncertainty metrics, are commonly used in image-based analysis as they are easier for nondevelopers to understand. Clinicians can then make informed decisions on whether to accept or reject the predictions based on their knowledge and judgment. However, few doctors are currently aware of how to review the technical aspects of AI tools. Therefore, it is essential that all information in training materials is presented in an understandable way, using methods and metrics that are familiar to health care professionals, and that professional associations are involved as institutional stakeholders.

Medical AI technologies should include alert systems to warn users when cases fall “out of scope” or outside the tool’s “intended use,” ensuring the tool is not applied to patients it was not designed for, thereby preventing potential harm. For instance, if an AI system for breast cancer screening was primarily trained on elderly patients with low-density breasts, its performance may be inferior for young patients with high-density breasts. Doctors should be informed of these limitations during training and should be alerted when the system is used in these cases. In this regard, AI tools in oncological imaging must meet the AI Act’s high-risk requirements, including transparency about dataset limitations, essential for building trust among clinicians. Similarly, genomics tools such as polygenic risk scores [13] and biomarkers based on multiomics data [78,79] raise concerns about bias and generalizability, and in line with the obligations for documentation and human oversight of the AI Act, these risks should be addressed, and trustworthy use in clinical practice should be supported.

The additional training for medical professionals in the use of AI should include methods for quick and reliable assessment of tools in daily practice. This will enhance technical literacy in health care, demystify AI’s potential, and clarify the tools’ technical capabilities, thereby increasing trust. Nonetheless, the required training should be sufficient for safe use but limited in terms of attention and time consumption. AI-driven software, as another set of tools in daily practice, should integrate seamlessly into workflows without causing major interruptions. Doctors should be able to focus on patient care and medical knowledge rather than learning AI’s inner workings. Therefore, adequate education and training at the time of AI-tool implementation, along with clear documentation, explainability, uncertainty metrics, and alert systems, are essential for building trust in AI solutions among health care professionals and other users.

## Situating Humans: Human-in-the-Loop, Human in the Center, Humans in Loops of Trust

Approaches from the ethics of AI perspective emphasize that AI should involve human features, that humans need to be in the loop of AI processes, or that humans need to have the last word in decision-making to ensure the trustworthiness of an AI system [80]. According to the EU AI Act, AI should be human-centric, and thus, it should involve human-in-the-loop approaches that ensure the involvement of humans in all stages of medical AI development and testing. As mentioned earlier, trust is related to several interpersonal relationships and institutional conditions, and in this case, also to automated or autonomous technologies. Therefore, rather than asking *how* humans can be integrated into the AI loop, the situatedness of humans in terms of where and when within the entanglement of social-technological and institutional relations in connection to trust should receive further attention.

Workshop outcomes emphasize that a tool’s trustworthiness is established through trust in humans (and their expertise), such as developers or physicians, and relationships. Moreover, trusting relationships are framed by trust in the social system or institutional framework in which it is embedded. Therefore, trust can be considered a dynamic process involving several actors and interactions among individuals, technologies, and institutions. Tackling issues around trust in medical AI necessitates emphasizing its multifaceted character, situatedness, and contextuality. In this regard, trust evolves as a dynamic process, spanning machine reliability, human relationships, and broader scientific skepticism. In addition, it also has a temporal dimension, as past experiences significantly influence perceptions of AI reliability in medicine.

In all its complex multidimensionality, which was discussed during the workshop, trust places humans at the center with the claim that we trust people, not just tools. Tools are judged on their reliability and credibility. For instance, an AI tool is credible and reliable when the system functions in a way that the individual expects and expectations themselves are cascading, considering that the designer, producer, the user (eg, medical doctor or the patient) may have different expectations that can be tied to each other as well as their own situatedness. The integration of AI into real-world medical settings relies on its promised functionality, such as accurate disease prediction or classification. This includes defining features, protocols, and patient-centered considerations during the design and implementation phases, including quality control after implementation. Ultimately, trust is built by humans, such as developers and physicians, who collaborate in increasingly multidisciplinary and international teams is also reenacted in settings that cannot be completely predefined and restricted.

Various examples of AI tools used in clinical settings allow insights into how human is situated. For instance, against a rapidly expanding plethora of AI tools, the clearance of these by the Food and Drug Administration in the US context relies on categorization of tools as low-risk and high-risk [81]. In the clinical context, however, many factors impact how risks are understood in practice, such as whether the tool is used to assist with a process under the clinician’s complete control, such as a tool assisting a cardiologist with drawing the contours of the heart for consultation. On the contrary, there are also cases where the clinician must assume the reliability of tools, where institutional structures, such as health ministries and researchers, have already considered potential biases of the datasets that informed the algorithm, as well as during the entire development, validation, and auditing process. While the algorithmic impact assessment is expected to be performed by other experts before tools make it to clinical practice, the consequences of the opposite could be drastic for the patients and doctors: for instance, a tool that does not consider the breast glandular tissue of individuals according to varying lifestyles and demographics may directly impact the outcome of diagnosis or intervention.

The integration of AI tools into human medical professionals’ practices presents a challenge as AI systems and medical professionals are intrinsically different in terms of the registers that they rely on. AI systems rely on mathematical rules, functions, and statistical algorithms, enabling their performance to be assessed with mathematical calculability. However, a major concern regarding the credibility of AI in medicine is the accurate translation of these mathematical capabilities into meaningful clinical value. One aspect that is relevant here is accuracy. The possible expectation for health care professionals to verify all AI-driven recommendations as an additional task to their daily routine to avoid harm in some patients, rather than only those flagged as potentially erroneous, would pose a significant impediment to the trustworthiness and widespread adoption of these technologies as standalone tools without human oversight.

The balance between sensitivity and specificity is particularly challenging in medical domains, such as oncology. Screening tools, like those used for breast cancer detection, where the prevalence of cancer in the examined samples is low, require high sensitivity to ensure that all potential cases are identified, even at the expense of a higher rate of false positives. The sensitivity of the AI tool should at least match, if not exceed, the average sensitivity of radiologists in national screening programs [82]. In such scenarios, the false-positive results can be addressed through subsequent verification procedures, such as biopsies. While the cost of a false positive result may be less harmful than missing the cancer, it can still have a significant impact on patients receiving misleading information [83], exemplifying the multidimensionality of AI’s use. Conversely, in applications, such as those aimed at distinguishing small liver tumors from other lesions, high specificity is essential for early and accurate diagnosis, enabling timely treatment and improved patient outcomes.

The question, therefore, is: what level of accuracy and precision would be deemed “good enough” for specific clinical applications to foster trust and drive the wider adoption of AI tools in medicine? Addressing this challenge requires human understanding and judgment of the trade-offs among sensitivity, specificity, and the practical implications for health care professionals and patients.

## Conclusions

This article centers on a synthesis of multidisciplinary discussions on trustworthy AI during an expert workshop, which allows us to underscore the complexities surrounding this topic in the evolving landscape of medical technology. The findings highlighted the nexus of knowledge on trust, emphasizing the situatedness of the human. Thus, conceptualizing and building trustworthiness in AI requires a comprehensive, multifaceted approach.

Trust and trustworthiness are not legal concepts. However, trustworthiness can be a feature of an AI system, and trust can be a purpose and effect of a law. Such trust can be reached by providing standards for trustworthy AI, including (1) transparent rules on roles, responsibilities, and procedures for AI development and by enforcing those rules through liability norms as well as through clear consequences for violation of duties; (2) quality and security features for AI tools; and (3) opportunities for public participation and debate. Clear, well-implemented, and effectively enforced laws contribute to public trust. Therefore, AI laws should prioritize defining roles, procedures, responsibilities, and liabilities alongside establishing efficient systems for control and enforcement. For example, the EU AI Act focuses on trustworthiness to foster trust through regulation [84]. Our article demonstrates that scholars from various domains converge on the understanding that achieving trust in AI is complex and involves individuals, making this legal expectation a desirable mission reflected in global AI regulations.

Contrary to the “move fast, break things” innovation maxim, setting high standards for quality and security is essential to maintaining the reliability and integrity of AI tools. Trust in AI is not an inherent feature, but a belief held by users. Trust involves the procedures, steps, and individuals behind the creation, use, and maintenance of AI tools, including developers, health care professionals, and update teams. Therefore, AI developers must build reliable systems to earn public trust and uphold their reputation. User trust relies on the AI’s trustworthiness, which depends on the transparency and verifiability of its development processes. To incorporate elements of human oversight by design, developers, for instance, strive more and more to visualize explainability in user interfaces when users interact with AI [85].

Historically, medicine embraced the authority of individual experts. In the 20th century, the concept of the “eminent expert” was replaced by evidence-based medicine (EBM) [86], which relies on scientific evidence produced by the broader scientific community. EBM establishes trust in new diagnostic and treatment methods. Similarly, the credibility of medical AI tools will grow with increasing good-quality evidence and defined accuracy and precision levels needed for specific tasks.

Researchers in the field of ethics of AI in medicine must strive for accuracy and precision by providing clear definitions for concepts, such as trustworthiness or trust in specific contexts, and situating them within broader societal issues. It is crucial to analyze the complex relationship between trustworthiness, trust, and explainability [87,88] and to find out what kind of explanations are required for specific situations and applications in medicine to adapt procedures accordingly. Interdisciplinary research, involving social scientists and clinicians, is crucial to incorporating clinical concepts [89]. This interdisciplinary approach involves a layered understanding, where ethical, societal, and legal issues from AI and clinical applications add further risks and complexities when combined [13].

Experts highlighted that trust in AI systems is bolstered not only by understanding how these tools operate but also by ensuring a balance between knowledge and acceptable uncertainty. Users need confidence that AI functions as intended.

Explainability can also play a role in the users’ and patients’ right to know, which affects their autonomy and agency to make informed decisions [90,91]. Transparency is crucial; however, our findings highlight the importance of providing context-specific, stakeholder-relevant information. Failure to uphold this right can result in epistemic injustices [92-95], especially for marginalized groups, who may be denied knowledge that may affect their rights and well-being. Upholding the right to know helps prevent epistemic injustices and enhances patients’ ability to comprehend the rationale behind diagnoses, treatment recommendations, and the use of AI tools in their care.

Our findings emphasize that establishing AI trustworthiness in health care requires a robust, human-centered, multistakeholder approach. Historically, AI tools have been predominantly engineered with limited input beyond technical development teams, often with minimal involvement from health care professionals. Documented real-world examples that fulfill all the requirements for trustworthy AI discussed in this article are scarce, and most scientific research focuses on evaluating some dimensions of trustworthiness, for example, by providing methodology to assess AI tools in diverse populations [96]. It is worth noticing that major private AI companies do report involving ethics and safety teams as part of their development, with parallels to our discussion, including techniques such as the use of external red-teaming to ensure privacy and safety, stakeholder engagement, and frameworks to assess biases and other social and ethical risks (for instance, Google DeepMind’s three-layered framework for evaluating the social and ethical risks of AI systems). However, the fact that many of the state-of-the-art AI models still remain black-box in terms of their inner workings and training data poses a challenge to their wider use in health care. In this regard, several engagement techniques can be used to collect adequate insights and continuous feedback from stakeholders. It is crucial that engagement activities are diverse and inclusive to ensure the AI tool is designed for all and to promote equity in AI-mediated health care.

Engaging only stakeholders with high education and digital proficiency may result in tools that are inaccessible to individuals with low digital literacy, which may impact health outcomes [97]. Including vulnerable groups and minorities in AI design and development can help develop tools that are respectful of diverse needs and contexts. Hence, we recommend that AI development teams should involve all relevant stakeholders at all stages of the AI life cycle, that is, conception, technical development, clinical validation, and real-world deployment (Textbox 1).

**Authors’ recommendations for involving relevant stakeholders across all stages of the AI (artificial intelligence) life cycle.**

The authors provide recommendations for involving relevant stakeholders at each stage of the AI life cycle, emphasizing continuous engagement to ensure transparency, accountability, and inclusiveness throughout the development and deployment of AI systems.

**AI design phase**:

Depending on the AI application, a range of health care professionals, such as general practitioners, specialists (domain experts), health care managers, nurses, and technicians, should be engaged for requirements elicitation. This includes defining the intended use of AI tools, clinical endpoints, success criteria, and the specific requirements for trustworthiness and transparency. Furthermore, clinicians can help specify the most adequate approaches for explainability, the types of explanations needed, and the conditions under which alerts or warnings should be issued.

Beyond clinicians, patient engagement is vital for identifying user needs, preferences, and potential barriers to trust and adherence to AI-mediated care. Ethicists and social scientists should also play a crucial role during conception, especially for anticipating the application-specific ethical and social impacts of the AI tools, such as misalignments with fundamental rights, effects on deskilling, changes in power relationships, and alterations in human behaviors as AI is integrated into care settings. Considering the approaches of trustworthy AI in Table 1, they can also apply qualitative research methods to examine the intentions and actions of the AI developers, understand the AI-mediated doctor-patient relationship, and assess the trustworthiness and perspectives of the institutions involved.

**AI development phase**:

At this stage, the AI team should focus on translating the stakeholder-defined requirements and identified risks into development strategies, including mitigation measures. This includes compiling diverse and representative training datasets, employing machine learning methods that minimize potential biases, and developing AI-human interfaces that enhance user interaction and comprehension of the AI system. It is also important to ensure that the AI tool’s technical development considers existing care models for seamless integration into real-world practice, thereby adding value without disrupting established workflows. During the development phase, it is important that stakeholders continue to be engaged, so they can monitor the technical developments and provide continuous feedback on the AI tool’s anticipated level of trustworthiness.

**AI validation phase**:

During the validation phase, it is important to assess the AI tool’s trustworthiness across multiple dimensions, including robustness under real-world conditions, level of transparency and explainability, fairness concerning diverse groups, usability in practice, and ethical and social compliance. This phase should continue to engage all stakeholders, including social scientists to evaluate the socio-behavioral implications of the AI tools on end users, such as whether the tools and their explanations enhance or diminish user confidence and trust, affect users’ ability to retain judgment when using the AI tool, and improve or degrade doctor-patient relationships.

**AI deployment phase:**

Once the tool is validated, certified, and deployed, a multistakeholder team must continue to monitor its performance and impact in real-world settings. This includes conducting periodic evaluations and audits to identify any performance degradation or emerging ethical issues, implementing logging systems to enhance traceability and accountability, and ensuring robust human oversight mechanisms are in place. These steps are crucial for maintaining user trust and ensuring that human autonomy is respected, demonstrating that there is adequate governance surrounding the use, maintenance, and oversight of the AI tool.

By adhering to a human-centered, multicentered framework, AI development teams can create tools that are not only technically efficient but also ethically sound and broadly accepted by all relevant users, groups, and institutions. This inclusive approach ensures that AI systems are developed with an in-depth understanding of the various contexts in which they will operate, leading to more effective and equitable AI-mediated health care.

Recent years have witnessed a surge of foundation models, that is, AI models trained with massive data and computational resources, which can also solve medical problems out-of-the-box. Though not the focus of this article, the aspects of trust discussed here can also be relevant when pretraining foundation models or fine-tuning them for deployment in health care use cases with additional local data. Studies focusing on the trustworthiness of foundation models have started to emerge [98], and we expect more work in this direction in the future.

Finally, future research should examine how the legal notion of “trustworthy AI” under the EU AI Act is interpreted and implemented across different member states, especially in clinical contexts. Comparative legal analysis could reveal how national competent authorities enforce trust-related obligations, such as human oversight or transparency in medical AI systems. Furthermore, empirical studies are needed to assess whether regulatory compliance translates into perceived trust among clinicians and patients. For instance, guidelines and recommendations, whether they have been established top down by policy makers or bottom up by the scientific community and practitioners, need to be tested to see if they are the effective and practical governance tools they are intended to be. This requires an interdisciplinary effort by asking if their uptake by individuals as well as institutions is meaningful in practice. Consider, exemplarily, the assessment of the added value of the inclusion of patient and citizen groups in the design, validation, and deployment phases of AI systems and the development of standardized frameworks for measuring trust across stakeholders and exploring how such trust is cultivated or undermined over time. These also involve evaluating the various roles of stakeholders and experts in these processes, particularly with regard to responsibility, distribution of roles, and power. To determine whether trust-building is effective, new methodologies will be required and should go beyond the quantitative measurement of key performance indicators and allow for thorough qualitative assessments that allow agile management.

## Abbreviations

AI: artificial intelligence
AI HLEG: High-Level Expert Group on Artificial Intelligence
EBM: evidence-based medicine
EU: European Union
FUTURE-AI: Fairness, Universality, Traceability, Usability, Robustness, and Explainability–artificial intelligence
MDR: Medical Device Regulation
STS: science and technology studies
WHO: World Health Organization
